# Human dimethylarginine dimethylaminohydrolase 1 inhibition by proton pump inhibitors and the cardiovascular risk marker asymmetric dimethylarginine: *in vitro* and *in vivo* significance

**DOI:** 10.1038/s41598-017-03069-1

**Published:** 2017-06-06

**Authors:** S. Tommasi, D. J. Elliot, J. A. Hulin, B. C. Lewis, M. McEvoy, A. A. Mangoni

**Affiliations:** 1 Department of Clinical Pharmacology, School of Medicine, Flinders University and Flinders Medical Centre, Adelaide, Australia; 2 Flinders Centre for Innovation in Cancer, School of Medicine, Flinders University, Adelaide, Australia; 3 Centre for Clinical Epidemiology & Biostatistics, Hunter Medical Research Institute, University of Newcastle, Newcastle, Australia

## Abstract

Proton pump inhibitor (PPI)-induced inhibition of dimethylarginine dimethylaminohydrolase 1 (DDAH1), with consequent accumulation of the nitric oxide synthase inhibitor asymmetric dimethylarginine (ADMA), might explain the increased cardiovascular risk with PPI use. However, uncertainty exists regarding whether clinical PPI concentrations significantly inhibit DDAH1 under linear initial rate conditions, and whether PPI-induced DDAH1 inhibition significantly increases ADMA in humans. DDAH1 inhibition by esomeprazole, omeprazole, pantoprazole, lansoprazole and rabeprazole was determined by quantifying DDAH1-mediated L-citrulline formation *in vitro*. Plasma ADMA was measured in PPI users (n = 134) and non-users (n = 489) in the Hunter Community Study (HCS). At clinical PPI concentrations (0.1–10 μmol/L), DDAH1 retained >80% activity vs. baseline. A significant, reversible, time-dependent inhibition was observed with lansoprazole (66% activity at 240 min, P = 0.034) and rabeprazole (25% activity at 240 min, P < 0.001). In regression analysis, PPI use was not associated with ADMA in HCS participants (beta 0.012, 95% CI −0.001 to 0.025, P = 0.077). Furthermore, there were no differences in ADMA between specific PPIs (P = 0.748). At clinical concentrations, PPIs are weak, reversible, DDAH1 inhibitors *in vitro*. The lack of significant associations between PPIs and ADMA in HCS participants questions the significance of DDAH1 inhibition as a mechanism explaining the increased cardiovascular risk reported with PPI use.

## Introduction

Proton pump inhibitors (PPIs) are widely prescribed for the treatment of disorders characterized by excessive gastric acid production^1^. Globally, PPIs account for 113 million prescriptions and USD13 billion in sales per year^2, 3^. In 2009, 84 million visits with documented PPI use were observed in the US ambulatory setting alone^4^. However, there is increasing evidence that PPIs are inappropriately prescribed, particularly in the older population; an area of concern due to an emerging and ever-growing list of adverse effects linked to PPI use^5^. Recent studies have reported associations between long-term PPI use and adverse clinical outcomes, such as incident chronic kidney disease, end-stage renal disease, falls and fractures, dementia, infections, and vitamin and mineral deficiency^6–10^. In addition, PPI use has been associated with an increased risk of adverse cardiovascular events, yet the exact mechanism mediating this effect has been largely unassessed^11, 12^. One proposed mechanism involves asymmetric dimethylarginine (ADMA), a potent endogenous inhibitor of nitric oxide synthase (NOS). There is strong evidence that increased plasma/serum ADMA concentrations independently predict adverse cardiovascular outcomes via an impairment of nitric oxide (NO)-mediated processes such as endothelial-dependent vasodilation and regulation of vascular tone and blood pressure^13^. Ghebremariam *et al*. recently reported that PPIs exhibit a class effect to competitively inhibit isoform 1 of the enzyme dimethylarginine dimethylaminohydrolase (DDAH1, IC_50_ = 51–63 µmol/L), which is responsible for the degradation of ADMA into L-citrulline and dimethylamine^14^. PPI-mediated DDAH1 inhibition was associated with increased intracellular ADMA concentrations and reduced NO synthesis in endothelial cells^14^. Furthermore, the PPI omeprazole impaired vascular reactivity *ex-vivo*, whereas subcutaneous administration of lansoprazole in C57BL/6 J mice increased systemic ADMA concentrations^14^. Despite the potential clinical relevance of these observations, the reported study had several limitations: the observed PPI concentrations in human studies (generally between 0.1–10 µmol/L)^15^ are considerably lower than the reported IC_50_ values and the concentrations of omeprazole used in vascular reactivity studies (100 µmol/L), the dose of lansoprazole used in C57BL/6 J mice (30 mg/Kg/day) are considerably higher than the typical doses in humans (15–30 mg/day in adults), the incubation conditions differed considerably from what is observed to be associated with linear conversion of ADMA into L-citrulline, no information was provided about the stability of PPIs in the experimental system, and any time-dependent effect of DDAH1 inhibition was not assessed^14^. The use of initial rate conditions in kinetic assays is important to meet the assumptions of the Michaelis Menten enzyme kinetic model. When investigating drugs as competitive inhibitors of enzyme activity it is essential to perform experiments using strictly linear initial rate conditions, and probe substrate concentrations must be equal to, or less than, the K_m_ values. This allows the quantitative comparison of the observed effects. The use of non-linear conditions, such as long incubation time, may result in experimental artefacts, namely the degradation of the substrate, the enzyme or other components in the incubation mixture, that adversely affect data interpretation^16^.

Furthermore, minimal information exists regarding whether PPI use is associated with significant elevations in serum/plasma ADMA concentrations in humans, and whether individual PPIs may exhibit differential effects.

Here, we sought to address these issues by (a) investigating the kinetics of human DDAH1 inhibition by five commonly prescribed PPIs (esomeprazole, lansoprazole, omeprazole, rabeprazole, and pantoprazole) under initial rate conditions with concentrations observed in human studies, (b) testing the hypothesis that PPI-mediated DDAH1 inhibition is time-dependent, which may be associated with the previously reported chemical instability of PPIs during *in vitro* studies^17^, and (c) measuring plasma ADMA concentrations in PPI users and non-users participating in an epidemiological cohort of human ageing, the Hunter Community Study (HCS).

## Results

### DDAH1 inhibition

**Figure 1:**
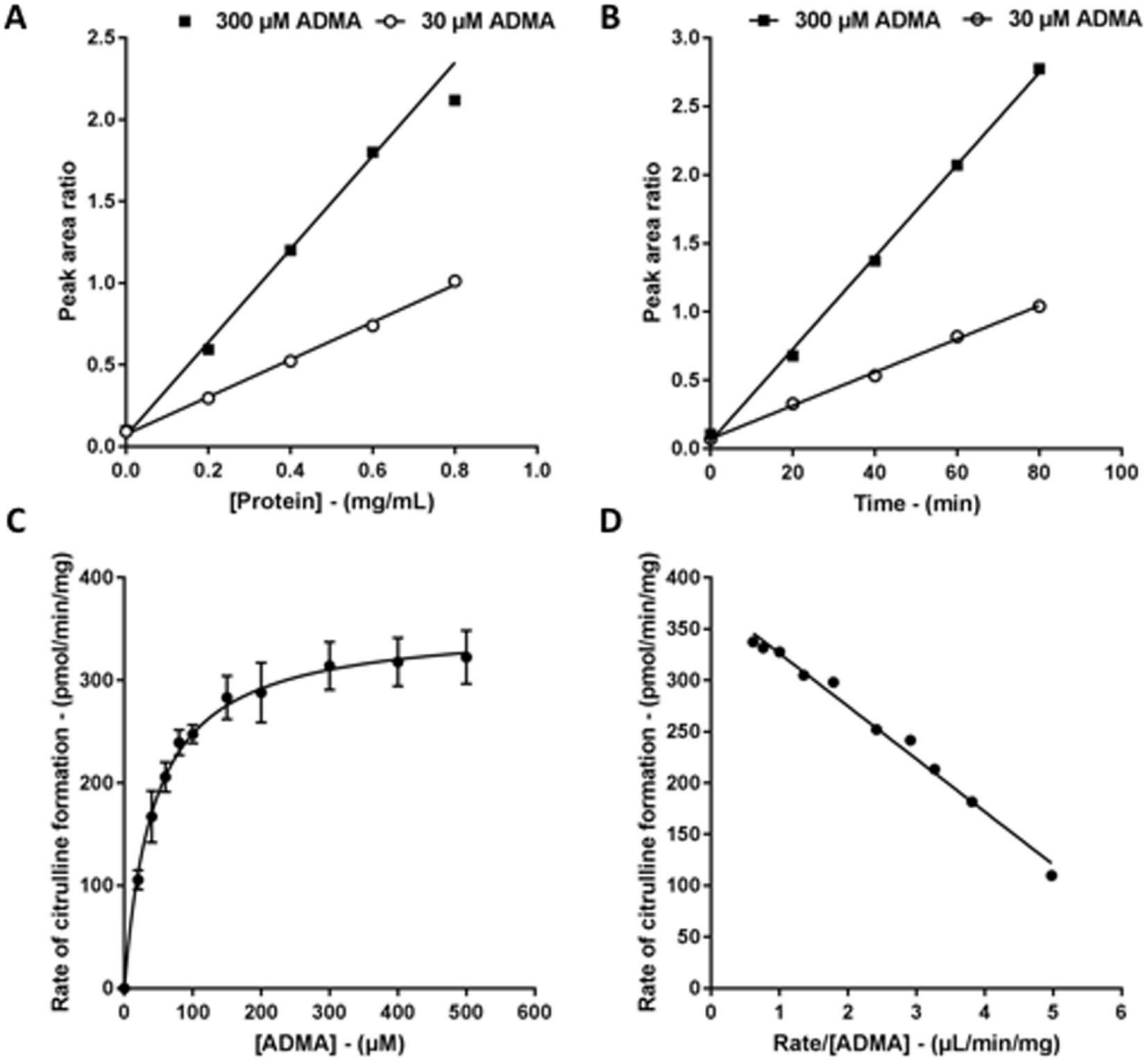
Kinetic plots representing the conversion of ADMA to L-citrulline by DDAH11. Protein linearity (**A**) and time linearity (**B**) data were collected in singlicate. For ADMA concentration versus rate (**C**) each data point is the mean of three singlicate experiments and error bars represent the standard error. The data is represented as an Eadie-Hoffstee transform in (**D**). The Michaelis-Menten fit is shown as a solid line in panel **C** and **D**.

Linear conditions for the conversion of ADMA into L-citrulline were observed up to 0.6 mg/mL and 80 min for protein concentration and time, respectively. Using 0.4 mg/mL protein and a 30-min incubation time, further experiments were conducted to characterize the kinetic behaviour of the DDAH1 expression system. ADMA conversion to L-citrulline resulted in *K*_m_ and V_max_ values of 45 ± 2 µmol/L and 352 ± 4 pmol/min/mg, respectively (Fig. 1 and Table 1). Therefore, the ADMA concentration used for inhibition studies was 45 µmol/L. Preliminary kinetic experiments were conducted in the presence and absence of EDTA at the concentration of 1 mmol/L. The addition of EDTA had no significant effect on enzyme kinetics (data not shown). Consequently, the *in vitr*o experiments conducted in this study did not include a chelating agent.

**Table 1:** Derived kinetic parameters for the conversion of asymmetric dimethylarginine to L-citrulline by dimethylarginine dimethylaminohydrolase 1.

Parameter	Replicate 1	Replicate 2	Replicate 3	Mean	SD
K_m_ (µmol/L)	49.8	45.3	41.5	45.5	4.2
V_max_ (pmol/min/mg)	378	355	326	353	26
F statistic	8307	4620	827		
R-squared	0.9990	0.9981	0.9892		

Kinetic constants (*K*_*m*_, V_max_) for L-citrulline formation were derived from fitting the Michaelis-Menten equation to experimental data using the nonlinear curve fitting software EnzFitter. SD: standard deviation.

#### Concentration-dependent effects

**Figure 2:**
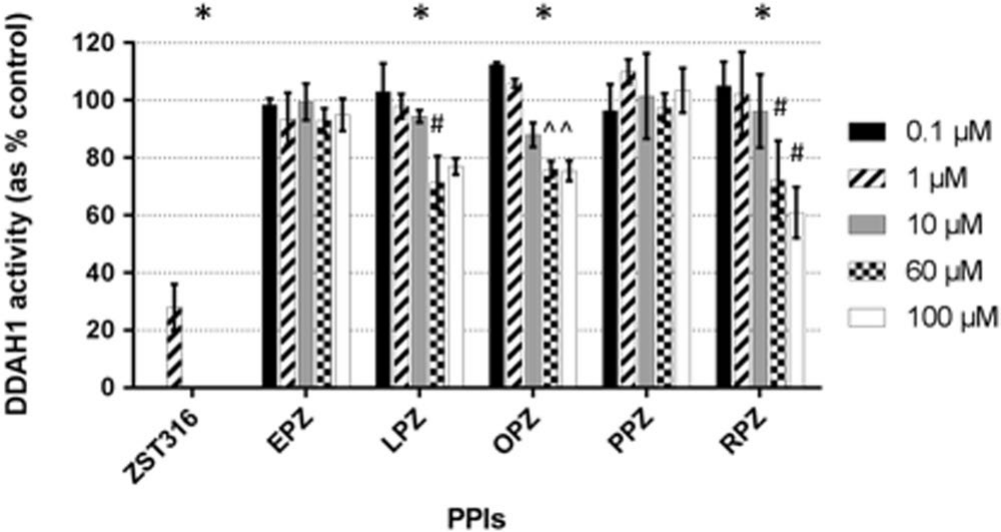
Concentration dependent inhibition of DDAH1 by ZST316 (positive control), esomeprazole (EPZ), lansoprazole (LPZ), omeprazole (OPZ), pantoprazole (PPZ) and rabeprazole (RPZ). Residual DDAH1 activity is reported as percentage of control activity. Each data point represents the mean of at least two single experiments. Error bars represent the standard deviation. *P < 0.01 for trend; ^#^P < 0.05 vs. baseline; ^^^P < 0.01 vs. baseline.

When tested under initial rate conditions, a concentration-dependent inhibition of DDAH1 was observed for lansoprazole, omeprazole and rabeprazole (P < 0.01 for trend), but not for esomeprazole or pantoprazole (Fig. 2). A significant inhibition relative to baseline was observed at concentrations of 60 µmol/L (lansoprazole and omeprazole) and 100 µmol/L (omeprazole and rabeprazole). As shown in Fig. 2, at concentrations of 100 µmol/L the inhibition of DDAH1-mediated formation of L-citrulline by lansoprazole, omeprazole and rabeprazole was 23%, 24%, and 39%, respectively. However, there was no significant DDAH1 inhibition at concentrations of 0.1, 1, and 10 µmol/L, with >80% of DDAH enzymatic activity retained for all PPIs studied (Fig. 2). Inhibition with the known DDAH1 inhibitor ZST316 was 72%^18^.

#### Time-dependent effects

The increase in incubation time from 30 to 240 min (60 µmol/L PPI concentration) was associated with a significant time-dependent inhibition, i.e. an increased inhibitory activity toward DDAH1, with lansoprazole and rabeprazole, but not with esomeprazole, omeprazole or pantoprazole (Table 2). DDAH1 inhibition ranged from 14% with pantoprazole to 75% with rabeprazole following a 240 min incubation (Table 2).

**Table 2:** Time dependent inhibition of dimethylarginine dimethylaminohydrolase 1 by proton pump inhibitors represented as percent residual activity.

PPI	30 min	60 min	120 min	180 min	240 min	P-value*
Esomeprazole	89 ± 10	86 ± 2	82 ± 10	79 ± 6	75 ± 9	0.253
Lansoprazole	93 ± 1	78 ± 14	71 ± 6	63 ± 8	66 ± 1	0.034
Omeprazole	87 ± 8	91 ± 20	78 ± 6	77 ± 4	76 ± 3	0.367
Pantoprazole	90 ± 10	89 ± 14	92 ± 13	92 ± 8	86 ± 7	0.959
Rabeprazole	56 ± 7	43 ± 5	31 ± 3	26 ± 6	25 ± 4	<0.001

Residual dimethylarginine dimethylaminohydrolase 1 activity (expressed as percentage of the control) following different pre-incubation times for each proton pump inhibitor. Each value represents the mean of three experiments ± SD. Experiments were performed with a proton pump inhibitor concentration of 60 µmol/L. PPI: proton pump inhibitor; *: for trend.

**Figure 3:**
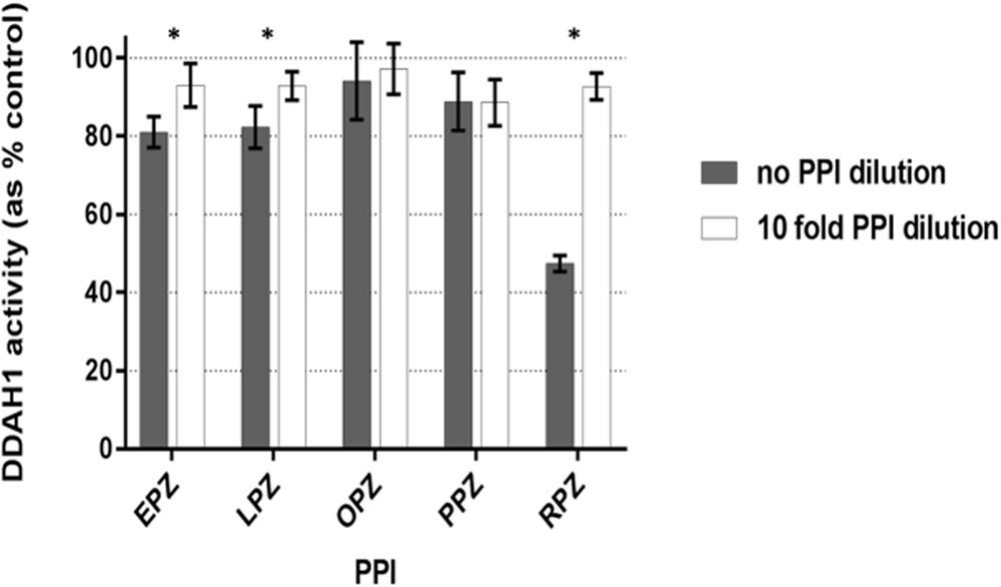
Reversibility of PPI binding to DDAH1. Measurement of DDAH1 activity is expressed as percentage of control activity (incubation with no PPI). Each data point represents the mean of two sets of triplicate experiments (10-fold PPI dilution) or one triplicate experiment (no PPI dilution). Error bars indicate the standard deviation. *P < 0.05; **P < 0.01.

To explore possible mechanisms for the observed time-dependent inhibition, particularly with rabeprazole, we investigated both the reversibility of the PPI-DDAH1 interaction and the stability and reactivity of PPIs during *in vitro* experiments. Reversibility was tested in a conventional two-step dilution experiment. DDAH1 activity was restored following a 10-fold dilution of the PPI concentration. This was particularly evident with rabeprazole, as it exhibited a greater magnitude of inhibition before dilution compared to the other PPIs (Fig. 3). These data suggest a reversible interaction between all PPIs tested and DDAH1.

**Figure 4:**
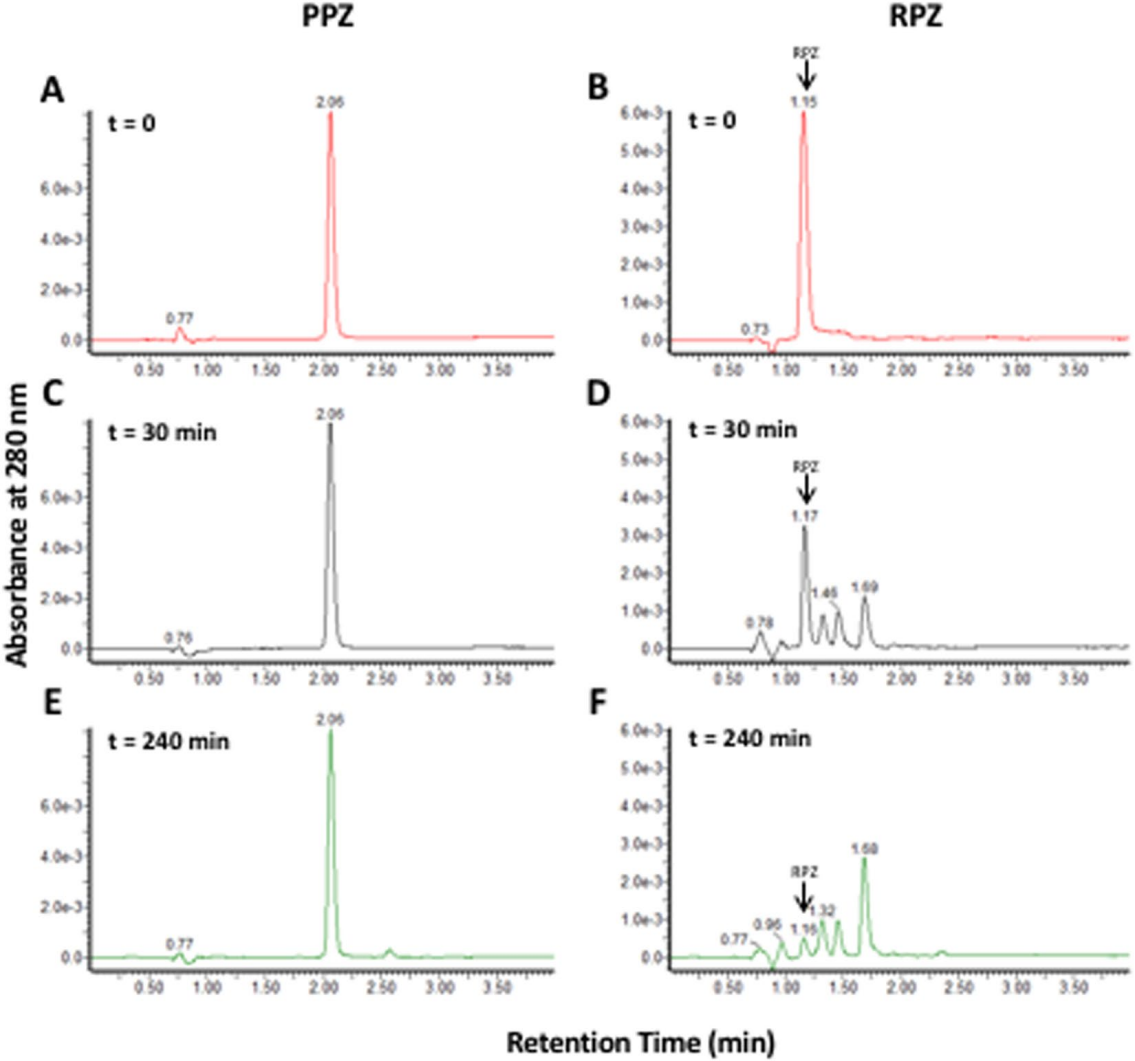
Detection of rabeprazole (RPZ) degradation products. UV chromatograms detected at 280 nm for pantoprazole (PPZ) and RPZ at time 0 (**A** and **B**, respectively), after 30 min of incubation (**C** and **D**, respectively) and after 4 h of incubation (**E** and **F**, respectively). Additional peaks in RPZ chromatograms are attributed to RPZ degradation products and were identified using published mass spectral data^17^ (data not shown). The arrow indicates the residual peak for RPZ.

We also observed a significant time-dependent degradation of rabeprazole (Fig. 4). Several degradation products were assigned based on previously published mass spectral data and the known breakdown of rabeprazole in neutral aqueous conditions^17^. Whilst rabeprazole exhibited a higher DDAH1 inhibition relative to other PPIs, its instability in 0.1 mol/L phosphate buffer at pH 7.4 leaves the mechanism of inhibition in these experiments unclear. These issues not withstanding, the data suggest that the *in vitro* rabeprazole-mediated DDAH1 inhibition is reversible. Furthermore, it is important to note that none of the PPIs tested reacted directly with ADMA, thus ruling out the possibility of substrate limitation as the cause of inhibition (data not shown).

Taken together, these data suggest that, at concentrations normally measured in humans, esomeprazole, lansoprazole, omeprazole, pantoprazole, and rabeprazole are mild, reversible, inhibitors of DDAH1 *in vitro*.

### PPI use and ADMA in the Hunter Community Study

Clinical and demographic characteristics of PPI users (n = 134) and non-users (n = 489) are described in Table 3. In unadjusted analyses, PPI users were significantly older, had a higher rate of previous myocardial infarction and prevalence of diabetes, used more statins and renin-angiotensin system inhibitors, and had higher triglyceride and ADMA concentrations compared to non-users. By contrast, PPI non-users had higher total cholesterol and LDL-cholesterol concentrations, diastolic blood pressure, and estimated glomerular filtration (eGFR) rate relative to users (Table 3). The PPI-user group consisted of 32 esomeprazole users, 40 omeprazole users, 23 rapebrazole users, 21 pantoprazole users, and 18 lansoprazole users. There were no significant differences in age and eGFR between users of different PPIs (Table 4).

**Table 3:** Clinical and demographic characteristics of the Hunter Community Study cohort.

Variable	PPI non-users (n = 489)	PPI users (n = 134)	P-value
Age (years)	63 (59–69)	66 (61–72)	<0.001
Females (%)	48.3	44.6	0.446
Myocardial infarction (%)	4.1	9.8	0.011
Stroke (%)	2.5	2.2	0.877
Diabetes or use of antidiabetic drugs (%)	10.0	18.0	0.010
Current smoker (%)	6.6	5.7	0.944
Regular alcohol consumption (%)	69.2	67.6	0.733
Statins (%)	31.5	42.9	0.017
Diuretics (%)	12.4	12.0	0.915
Beta-blockers (%)	18.7	24.8	0.126
RAS inhibitors (%)	39.8	52.6	0.009
Antiplatelet agents (%)	3.4	6.7	0.093
NSAIDs (%)	12.9	15.8	0.392
Fasting glucose (mmol/L)	4.8 (4.4–5.3)	4.9 (4.5–5.4)	0.173
Total cholesterol (mmol/L)	5.1 (4.4–5.9)	4.8 (4.2–5.5)	0.015
HDL-cholesterol (mmol/L)	1.3 (1.1–1.6)	1.2 (1.1–1.6)	0.485
LDL-cholesterol (mmol/L)	3.1 (2.5–3.8)	2.8 (2.3–3.5)	0.008
Triglycerides (mmol/L)	1.1 (0.8–1.6)	1.3 (0.8–1.8)	0.033
C-reactive protein (mg/L)	2.0 (1.2–3.7)	2.5 (1.1–5.1)	0.095
SBP (mmHg)	135 (124–146)	135 (122–145)	0.563
DBP (mmHg)	81 (72–87)	76 (71–84)	0.004
ADMA (µmol/L)	0.54 (0.49–0.60)	0.56 (0.51–0.63)	0.002
eGFR (mL/min/1.73 m^2^)	80 ± 16	76 ± 17	0.005

PPI: proton pump inhibitor; RAS: renin-angiotensin system; NSAIDs: non-steroidal anti-inflammatory drugs; HDL: high-density lipoprotein; LDL: low-density lipoprotein; SBP: systolic blood pressure: DBP: diastolic blood pressure; ADMA: asymmetric dimethylarginine; eGFR: estimated glomerular filtration rate (Modification of Diet in Renal Disease formula).

**Table 4:** Plasma asymmetric dimethylarginine concentrations in users of specific proton pump inhibitors

	Esomeprazole (n = 32)	Omeprazole (n = 40)	Rabeprazole (n = 23)	Pantoprazole (n = 21)	Lansoprazole (n = 18)	P-value
Age (years)	69 (62–73)	68 (62–75)	63 (59–69)	65 (60–73)	66 (61–73)	0.092
eGFR (mL/min/1.73 m^2^)	77 ± 20	73 ± 15	76 ± 15	82 ± 20	74 ± 15	0.401
ADMA (µmol/L)	0.57 (0.52–0.64)	0.58 (0.51–0.64)	0.55 (0.50–0.65)	0.57 (0.51–0.60)	0.53 (0.49–0.63)	0.748

^e^GFR: estimated glomerular filtration rate (Modification of Diet in Renal Disease formula); ADMA: asymmetric dimethylarginine.

The following variables were associated (P < 0.2) with ADMA concentrations and were entered into regression analysis: age, gender, history of myocardial infarction and stroke, diabetes, regular alcohol consumption, use of statins, beta-blockers, diuretics, renin-angiotensin system inhibitors and antiplatelet drugs, total, HDL and LDL-cholesterol, triglycerides, C-reactive protein, and eGFR. In adjusted multivariable regression analysis, PPI use was not independently associated with ADMA concentrations (beta 0.079, 95% CI −0.001 to 0.025, P = 0.077; Supplementary Table S2). Furthermore, there were no significant differences in ADMA concentrations between users of the five PPIs studied (P = 0.748 for trend, Table 4).

## Discussion

We assessed the DDAH1 inhibitory activity of five widely prescribed PPIs (esomeprazole, lansoprazole, omeprazole, pantoprazole and rabeprazole) under strict linear conditions *in vitro*, using PPI concentrations normally observed in human plasma (0.1–10 µmol/L). All PPIs studied displayed weak, reversible, DDAH1 inhibition, albeit at concentrations greater than that observed clinically (60–100 µmol/L). A time-dependent inhibition was observed with lansoprazole and rabeprazole, with their inhibitory effect increasing with longer incubation time. Although the DDAH1 inhibitory potential of rabeprazole was greater than all other PPIs studied its level of inhibition still remained mild at clinically relevant concentrations. Our *in vitro* data support the lack of independent associations between PPI use and ADMA concentrations in an epidemiological cohort. Furthermore, there were no significant differences in ADMA concentrations with specific PPIs, including rabeprazole, the PPI we found to have the greatest DDAH1 inhibitory potential *in vitro*.

Recent studies have reported associations between PPI use and increased cardiovascular risk^11, 12^. PPI-mediated inhibition of the hepatic enzyme CYP2C19, essential for the activation of the antiplatelet prodrug clopidogrel, has been proposed as a potential mechanism behind this association. However, the increased cardiovascular risk with PPI use has been demonstrated to be independent of clopidogrel use^11^. In support of this, some PPIs displaying associations with cardiovascular risk are in fact relatively weak CYP2C19 inhibitors^19^. Recently, an alternative mechanism has been proposed to explain the cardiovascular toxicity: PPI-induced inhibition of the enzyme DDAH1 with a consequential increase in ADMA concentrations^14^. There is very good evidence that elevations in plasma ADMA concentrations independently predict cardiovascular morbidity and mortality, through inhibition of endothelial NO synthesis resulting in endothelial dysfunction and increased risk of cardiovascular events^13, 20^. The majority of ADMA is hydrolytically degraded by DDAH^21^, and as such a likely cause of an elevation in plasma ADMA concentrations is an impairment to DDAH function. A reduction in DDAH1 catalytic activity and/or expression is therefore a potential contributor to endothelial dysfunction and increased cardiovascular risk.

PPI administration was recently found to be associated with an inhibition of DDAH1 activity accompanied by increased ADMA concentrations both *in vitro* and *ex-vivo* and in animal models^14^. However, this study employed sustained incubation times (4 h) and utilized ADMA concentrations that correlated with the enzyme maximal rate (V_max_) rather than the substrate concentration at half maximal velocity (*K*_m_). Additionally, the concentrations of omeprazole in vascular reactivity studies (100 µmol/L) were significantly higher than those observed in humans (0.1–10 µmol/L). Similarly, the doses of lansoprazole used in C57BL/6 J mice (30 mg/Kg/day) were considerably higher than the typical doses in humans (15–30 mg/day in adults)^14^. Furthermore, the lack of assessment of PPI stability in recent studies suggests the observed PPI-mediated DDAH1 inhibition *in vitro* may be an artefact of the experimental conditions used.

We aimed to minimise the aforementioned limitations by investigating PPI-mediated DDAH1 inhibition *in vitro* employing a highly sensitive and specific UPLC-MS method to measure L-citrulline formation from ADMA. This method is characterized by high specificity and precision and does not require intensive sample pre-treatment or the use of an artificial substrate^22^. Kinetic characterization of DDAH1-mediated ADMA conversion to L-citrulline resulted in *K*_m_ and V_max_ values of 45 ± 2 µmol/L and 352 ± 4 pmol/min/mg, respectively. Our data are in agreement with the previously reported kinetic characterization of L-citrulline formation from ADMA by DDAH1 expressed in *E. coli*, obtained by a colorimetric assay^23^ (*K*_m_ = 68.7 μmol/L and V_max_ = 356 pmol/min/mg). However, the *K*_m_ data reported by Ghebremariam *et al*. exhibited an approximate 3–4 fold reduction in ADMA affinity (*K*_m_ = 180 μmol/L)^24^, which might be indicative of the aforementioned differences in the experimental conditions and the methods used to characterize DDAH1 activity *in vitro*.

Perhaps more importantly, observed C_max_ and area under the concentration-time curve (AUC) data following typically prescribed PPI doses in humans range between 0.2–23.2 µmol/L and 0.58–13.5 µmol/hr/L, respectively^15^, and are considerably lower than the inhibitory concentrations identified in previos studies (IC_50_ = 51–63 µmol/L)^14^. In order to obtain more clinically relevant data, we tested the inhibitory potential of PPIs within a concentration range of 0.1–10 µmol/L. Interestingly, no significant inhibition of DDAH1 activity was observed within this concentration range, suggesting that, at clinically observed concentrations, PPIs are unlikely to cause a significant inhibition of DDAH1 that would result in elevated ADMA concentrations. However, higher PPI concentrations in extravascular compartments cannot be ruled out. For example, PPIs can accumulate 1,000-fold in the secretory canaliculus of the parietal cell because of the favourable acidic environment; here PPIs irreversibly bind and inhibit H^+^, K^+^-ATPase transporters^15^. Nonetheless, there is currently no reported evidence for PPI accumulation within any other cellular compartment or under neutral/slightly alkaline pH conditions.

In contrast to previous studies^14^, we employed a PPI incubation time of only 30 min instead of the considerably longer 4 hours. However, a progressive increase in the incubation time in our study (up to 4 h) demonstrated the presence of a time-dependent inhibitory effect, with an increase in DDAH1 inhibition ranging from 29% to 61%. This effect was significant with lansoprazole and, especially, rabeprazole, but not with esomeprazole, omeprazole, or pantoprazole (Table 2 and Supplementary Figure 3). These data suggest that prolonged exposure to PPIs, particularly rabeprazole, might result in significant DDAH1 inhibition. The reversibility of PPI-binding to DDAH1 was verified following a 10-fold dilution of PPI concentration. The significant reduction in the resulting DDAH1 inhibitory potential demonstrated a displacement of the inhibitor from the DDAH1 active site, and thus reversibility. These data are consistent with previously reported observations^14^.

As there was no evidence for a PPI class effect on DDAH inhibition we also investigated the stability of PPIs within our experimental system. The recovery of each PPI was measured at the end of a canonical incubation. Whilst the recovery of esomeprazole, lansoprazole, omeprazole, and pantoprazole approached complete recovery, there was a significant reduction in the concentration of rabeprazole recovered. Rabeprazole degrades in a phosphate buffered solution at pH 7.4, thus resulting in the increased formation of degradation products over time. The comparison of our MS spectrum extracted for each peak with data reported by Bahandi *et al*.^17^ confirmed the identification of each specific rabeprazole degradation product (Fig. 4). As discussed previously, rabeprazole displayed a markedly potent DDAH1 inhibitory potential, which was distinct from all other PPIs studied. Since rabeprazole is not chemically stable under the experimental conditions utilized, it is likely the observed increase in DDAH1 inhibition in incubations comprising rabeprazole is largely due to the degradation product(s). Alternatively, if the inhibition of DDAH1 activity is indeed due to the residual rabeprazole, it would suggest that this compound is a significantly more potent DDAH1 inhibitor than estimated. The exact mechanism of rabeprazole-mediated inhibition of DDAH1 remains to be elucidated.

The measurement of plasma ADMA concentrations in our epidemiological cohort provides further support for the *in vitro* data. There were no independent associations between ADMA and the use of PPIs, as a class, after adjusting for clinical, demographic and biochemical confounders. Although there was a trending (P = 0.077) association between PPI use and ADMA concentrations, the observed differences in median ADMA concentrations between PPI users and non-users (~0.02 µmol/L, Table 3) are unlikely to be significant in terms of cardiovascular risk^13^. Although the aforementioned *in vitro* studies showed a significantly higher DDAH1 inhibitory activity with rabeprazole, particularly after prolonged exposure, we did not observe any significant differences in ADMA concentrations in users of rabeprazole vs. other PPIs. Therefore, at a population level, the PPI-mediated inhibition of DDAH is unlikely to be of biological or clinical significance. Kruzelnicka *et al*. have recently reported a lack of significant differences in ADMA concentrations with PPI use in a smaller cohort of non-diabetic men (PPI users: n = 53, age 59 ± 11 years; PPI non-users: n = 75, age 56 ± 10 years) with stable coronary artery disease^25^. However, in this study ADMA concentrations were measured with a commercially available ELISA method, which is reported to overestimate ADMA concentrations by ≥20% and is matrix-dependent. Since plasma samples from a human population are characterized by a heterogeneous matrix, ELISA methods for the determination of ADMA concentrations are less reliable than mass spectrometry when the endpoint is to differentiate sample groups^26^. Furthermore, a number of factors potentially affecting DDAH1 activity and ADMA concentrations, particularly gender, renal function and concomitant medicines, were not accounted for in regression analyses^22, 25, 27^.

In comparison to previous reports, our study comprises rigorous kinetic characterization of DDAH1 inhibition by PPIs under strictly linear conditions. We assessed PPI-mediated inhibition at clinically observed concentrations, and evaluated alternate pre-incubation times in determining the magnitude of inhibition and the stability of PPIs during incubation. Furthermore, the biological and clinical significance of PPI-mediated DDAH1 inhibition was investigated by measuring ADMA concentrations in an epidemiological cohort of older adults with various co-morbid states and concomitant medications, after adjusting for clinical and demographic confounders affecting *per se* ADMA concentrations.

We used lysate from cells expressing recombinant DDAH1, rather than purified DDAH1, to more accurately mimic the complex cytosolic environment where DDAH1 catalysis occurs *in vivo*. While it may be possible that other substituents of the cell lysate may interfere with the PPIs, ADMA or DDAH1 itself, the complexity of the cell lysate mixture creates an *in vitro* environment that closely approximates intracellular physiological conditions.

A possible limitation of the study is that, similar to other pharmacoepidemiological studies, the evidence of a dispensed prescription, in this case a PPI, does not necessarily reflect the actual intake of the drug by the participant. Further limitations involve the cross-sectional nature of the epidemiological study, which does not permit establishment of a cause-effect relationship between PPI use and ADMA concentrations, and the fact that plasma ADMA concentrations do not necessarily reflect intracellular concentrations^28^.

In conclusion, the *in vitro* and *in vivo* results of our study question the significance of DDAH1 inhibition and ADMA elevation as a mechanism to explain the increased cardiovascular risk reported with PPI use. Conclusions drawn from comparisons between our data and the work of others suggest that *in vitro* inhibition of DDAH1 by PPIs is highly influenced by the experimental conditions and by the stability of PPIs over time. It is clear, however, that PPI use does not translate into significant increases in plasma ADMA concentrations at a population level. Further studies are warranted to identify the pathophysiological mechanisms responsible for the increased cardiovascular risk associated with PPI use.

## Methods

### DDAH1 inhibition *in vitro*

The experimental protocols for the assessment of DDAH1 inhibition *in vitro*, described below, were approved by the Institutional Biosafety Committee of Flinders University (IBC No. 2009-08).

#### Materials

Deuterated L-citrulline (L-citrulline-d6) was obtained from Sapphire Bioscience (Sapphire Bioscience, Redfern, Australia). High purity water was obtained using a MilliQ Synergy UV Ultrapure water system (Merck Millipore, Sydney, Australia). Acetonitrile (Liquid Chromatography-Mass Spectrometry, LC-MS, Grade), 2-propanol and formic acid (High Performance Liquid Chromatography, HPLC, Grade) were obtained from Merck Millipore (Merck Millipore, Melbourne, Australia). All other laboratory grade chemicals and reagents were purchased from Sigma-Aldrich (Sigma-Aldrich, Sydney, Australia). DDAH1 over-expression was performed as reported by Tommasi *et al*.^18^ A single batch of lysate prepared from DDAH1-expressing HEK293Tcells was used for all *in vitro* experiments.

#### Analytical Instrumentation

Concentrations of L-citrulline and individual PPIs were measured using an Aquity Ultra Performance Liquid Chromatography, UPLC, (Waters, Sydney, Australia) coupled to a tunable UV variable wavelength detector and a quadrupole time-of-flight (qToF) Premier high-resolution mass spectrometer (Waters, Sydney, Australia). The electrospray ionisation source was operated in positive ionisation mode. ToF data were collected in MS mode between 100 and 500 Da with an instrument scan time of 0.5 sec and inter-scan delay of 0.05 sec. Further details of the mass spectrometer parameters are provided in Supplementary Table 1. Instrument control, data acquisition and data processing were performed using Waters MassLynx version 4.1 software (Waters, Sydney, Australia).

#### DDAH1 activity assay

L-citrulline formation was determined at 37 °C in a total incubation volume of 0.1 mL using 12 × 75 mm borosilicate glass tubes. Incubation mixtures contained DDAH1-expressing HEK293T cell lysate (0.4 mg/mL total protein), phosphate buffer (0.1 mol/L, pH 7.4), PPI (0–100 µmol/L) and ADMA (0 to 500 µmol/L). The DDAH1 inhibitor ZST316 (compound 10a)^18^ was used as a positive control for DDAH1 inhibition at a concentration of 1 µmol/L. Following pre-incubation (0 to 240 min), reactions were initiated by the addition of substrate (ADMA). After 30-min incubation reactions were terminated by the addition of 300 μL 0.1% formic acid in 2-propanol and 10 µL of the assay internal standard (30 µmol/L L-citrulline-d6). The samples were vortex mixed (20 sec) and cooled on ice for 10 min prior to centrifugation (10 min, 18,000 × g) to precipitate the proteins. The supernatant (300 μL) was transferred to clean 12 × 75 mm borosilicate glass tubes and the solvent was removed by evaporation in a MiVac concentrator (T = 50 °C, P = 30 mbar, -OH programme, 25 min). The residue was redissolved in 125 μL of a 1:4 water/0.1% formic acid in 2-propanol mixture and a 3 μL aliquot was injected onto the UPLC column for analysis.

The PPI concentrations used in DDAH1 inhibition experiments included those normally observed (0–10 µmol/L) in human studies at therapeutic doses^15^. Higher (supra-physiological) concentrations (60 and 100 µmol/L) were also investigated in order to more comprehensively characterize the potential dose-response relationship between PPIs and DDAH1 inhibition.

#### UPLC-MS analysis of L-citrulline

L-citrulline was separated on a Waters ACQUITY UPLC BEH HILIC column (1.7 µm, 2.1 mm × 100 mm) with a gradient mobile phase containing 0.1% v/v formic acid and acetonitrile in water at a flow rate of 0.3 mL/min. Full details are provided in the Supplementary File. Selected ion chromatograms were extracted from the total ion chromatogram at m/z 176.10 → 159.10 and 181.13 → 165.12 corresponding to the fragments of L-citrulline and L-citrulline-d6, respectively. Calibration standards were prepared by spiking L-citrulline (0, 1, 2, 3, 4 and 5 µmol/L) into the incubation matrix. Calibrators were treated in the same manner as incubation samples and calibration curves obtained by plotting the peak area ratio L-citrulline to internal standard versus the standard concentration.

#### UPLC analysis of PPIs

Proteins were precipitated by addition of three volumes of ice-cold methanol to the reaction mixture followed by cooling on ice for 10 min, and then centrifugation (18,000 × g, 5 min). An aliquot of the supernatant was diluted 10-fold with mobile phase and 3 µL of the diluted sample was injected for analysis. Chromatography was performed on a Waters ACQUITY UPLC BEH C18 1.7 μm (2.1 × 100  mm) column with an isocratic mobile phase (0.1% formic acid and 28% acetonitrile in water) at a flow rate of 0.3 mL/min. The column temperature was maintained at 35 °C and each PPI was detected at 280 nm. Further details of the experimental conditions are provided in the Supplementary File.

### PPI use and ADMA in the Hunter Community Study

#### Study population

Study participants consisted of a cohort of community-dwelling subjects aged between 55–85 years residing in Newcastle (NSW, Australia). Participants were recruited from the Hunter Community Study (HCS), a population-based cohort study on human ageing^29^. Participants were randomly selected from the electoral roll and contacted between December 2004 and December 2007.

Participants completed two self-report questionnaires and which were returned upon their attendance at the HCS data collection centre, during which time several clinical and biochemical parameters were also assessed. Clinical assessment included a full physical examination and measurement of routine biochemical parameters including C-reactive protein (CRP), fasting lipids and glucose concentrations, and renal function. Consent to link personal information obtained during the study to data from Medicare Australia and local health databases was also sought. A further package of three self-reporting questionnaires, to be returned by reply-paid post, was given to each participant to complete at home. The questionnaires provided details on demographic and socioeconomic characteristics, nutritional assessment, medical and surgical history, medication exposure, tobacco use and alcohol consumption. Full details of the data collected are described elsewhere^29^.

The sample group of participants for this investigation (n = 623) was derived from the initial cohort by simple random sampling. A comparison of this sample with the entire cohort showed no significant differences with respect to a range of clinical, biochemical, socioeconomic, and behavioural factors (data not shown). The HCS was performed according to the Declaration of Helsinki. All procedures were approved by the local ethics committee of the University of Newcastle and the Hunter New England Area Health Service (NSW, Australia). Informed consent was obtained from each HCS participant.

#### Assessment of PPI exposure

Treatment with PPIs, both as a class and as specific agents, was determined by accessing data from the Pharmaceutical Benefits Scheme (PBS), a program of the Australian Government that provides subsidised prescription drugs to Australian residents^30^. Participants with at least one PPI prescription dispensed within 91 days before or after the date of blood sampling, performed by the Hunter Area Pathology Service within one week of their visit at the HCS data collection centre, were identified as PPI users.

#### ADMA measurement

Blood was collected in EDTA tubes and centrifuged at 4 °C and 3,000 g for 10 min to separate plasma, which was stored at −80 °C until analysis. ADMA was measured in a 0.1 mL aliquot of plasma by hydrophilic-interaction liquid chromatography and isotope dilution tandem mass spectrometry^31^. The intra- and inter-assay coefficients of variation (CV) were <15%.

### Statistical analysis

Results are expressed as means ± SD, medians and interquartile ranges, or frequencies as appropriate. Variables were tested for normal distribution by using the Kolmogorov-Smirnov test. Differences between groups were assessed by one-way ANOVA or Mann-Whitney U test. Associations between clinical and demographic variables and plasma ADMA concentrations were assessed by Spearman’s rank correlation coefficient. Non-normally distributed variables were log transformed. Clinical and demographic variables showing associations with either PPI use or ADMA concentrations (P < 0.2) were entered in multiple linear regression analysis, along with potentially confounding variables, to identify factors independently associated with ADMA concentrations. Multicollinearity was tested by measuring the tolerance and the variance inflation factor values for each analysis. Analyses were performed using IBM SPSS Statistics Version 23.0 (IBM Corp., Armonk, NY, USA). A two-sided P < 0.05 indicated statistical significance.

## Electronic supplementary material

Supplementary_file
