# Supplementary material for: Human dimethylarginine dimethylaminohydrolase 1 inhibition by proton pump inhibitors and the cardiovascular risk marker asymmetric dimethylarginine: in vitro and in vivo significance
Source: Sci Rep. 2017 Jun 6;7:2871. doi: 10.1038/s41598-017-03069-1 (PMC5460274; doi:10.1038/s41598-017-03069-1)
Supplement: Supplementary file 1 — Supplementary_file [file 41598_2017_3069_MOESM1_ESM.doc]

**Human dimethylarginine dimethylaminohydrolase 1 inhibition by proton pump inhibitors and the cardiovascular risk marker asymmetric dimethylarginine: *in vitro* and *in vivo* significance**

S. Tommasi, D.J. Elliot, J.A. Hulin, B.C. Lewis, M. McEvoy, A.A. Mangoni

**Materials**

Deuterated L-citrulline (L-citrulline-d6) was obtained from Sapphire Bioscience (Sapphire Bioscience, Redfern, Australia). High purity water was obtained using a MilliQ Synergy UV Ultrapure water system (Merck Millipore, Sydney, Australia). Acetonitrile (LC-MS Grade), 2-propanol and formic acid (HPLC Grade) were obtained from Merck Millipore (Merck Millipore, Melbourne, Australia). All other laboratory grade chemicals and reagents were purchased from Sigma-Aldrich (Sigma-Aldrich, Sydney, Australia).

DDAH1 expression was performed as reported by Tommasi *et al*. in 2015 ^18^. A single batch of DDAH1 expressed in HEK293T cells was used in all the experiments. Small aliquots of the lysate from this single preparation were stored at -20 °C to avoid repeated freeze thaw cycles.

10 mM stock solutions of ADMA and L-citrulline were prepared in purified water whereas PPI stock solutions (10 mM) were prepared in DMSO. These solutions were stored frozen at -20 °C. A 1 mg/mL stock solution of L-citrulline-d6 was prepared in aqueous 0.1% v/v acetic acid and stored at -80 °C. The working internal standard solution was prepared by diluting the 1 mg/mL L-citrulline-d6 stock solution with water to 30 µM final concentration. The working internal standard solution was stored frozen at -20 °C.

**Analytical instrumentation**

Citrulline and PPI analyses were performed on a Waters ACQUITY^TM^ Ultra Performance LC™ system coupled to an Aquity TUV variable wavelength detector and Waters Premier quadrupole time of flight (qToF) mass spectrometer (Waters, Sydney, Australia) fitted with an electrospray ionisation source operated in positive ionisation mode. Time-of-flight (ToF) data were collected in MS mode between 100 and 500 Da with an instrument scan time of 0.5 sec and inter-scan delay of 0.05 sec. The mass spectrometer parameters are shown in Table S1. Instrument control, data acquisition and data processing were performed using Waters MassLynx version 4.1 software (Waters, Sydney, Australia).

**Table S1.** **Mass spectrometer instrument settings.**

| **Instrument Parameter** | **Setting** |
| --- | --- |
| Capillary voltage (kV) | 3.5 |
| Sampling cone voltage (eV) | 20.0 |
| Extraction cone voltage (eV) | 5.0 |
| Source temperature (°C) | 90 |
| Desolvation temperature (°C) | 300 |
| Cone gas flow(L/Hr) | 30.0 |
| Desolvation gas flow (L/Hr) | 400.0 |
| Collision energy | 5.0 |
| Collision Cell Entrance | 2.0 |
| Collision Exit | -10.0 |
| Collision Gas Flow (mL/min) | 0.4 |

**UPLC-MS analysis of L-Citrulline**

L-citrulline was separated from the matrix components on a Waters ACQUITY UPLC® BEH HILIC column (1.7 µm, 2.1 mm x 100 mm) held at 35 °C. The mobile phase comprised acetonitrile containing 0.1% v/v formic acid (mobile phase A) and 0.1% formic acid in a solution of 10 % v/v acetonitrile in water containing 0.1% v/v formic acid (mobile phase B) at a flow rate of 0.3 mL/min. Initial conditions were 95% mobile phase A, 5% mobile phase B. The proportion of mobile phase B was increased linearly to 40% over 5 min then returned to 5% for 2 min to re-establish equilibrium before injecting the next sample for analysis.

Extracted ion chromatograms (EICs) were obtained with a mass window of 0.02 Da from total ion chromatogram (TIC) employing the m/z corresponding to the 176.10→159.10 and 181.13→165.12 fragments of L-citrulline and L-citrulline-d6 respectively. Calibration curves were obtained by plotting the peak area ratio L-citrulline to internal standard versus the standard concentration (Figure S1).

The method was assessed for linearity, reproducibility (intra-assay and inter-assay accuracy) and sensitivity (limit of detection or LOD and limit of quantification or LOQ). Calibration standards comprised of 6 L-citrulline concentrations (0, 1, 2, 3, 4 and 5 µM) spiked into the incubation matrix and were extracted and reconstituted in the same manner as incubation samples (Figure S2).


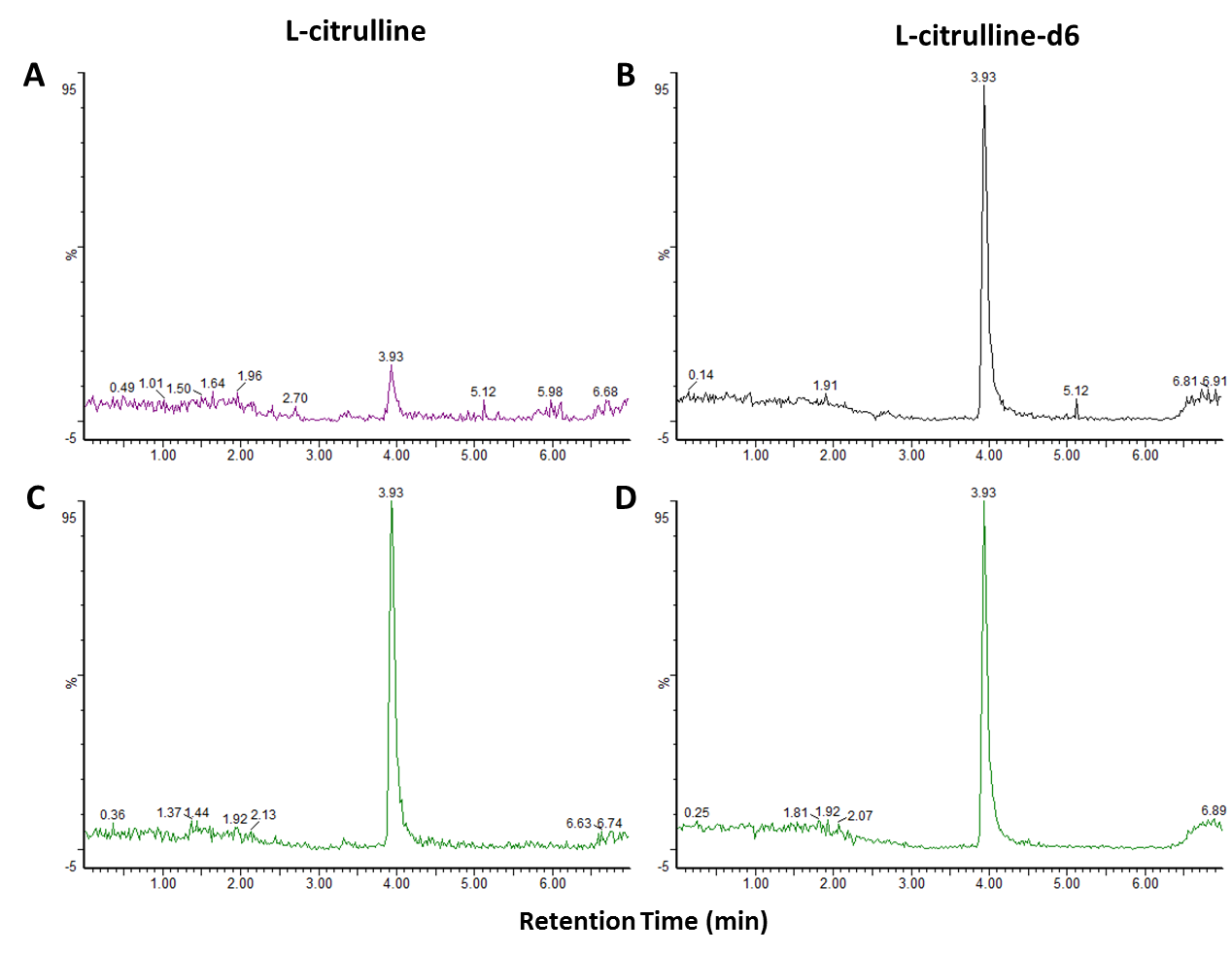


**Figure S1.**  Representative chromatograms for L-citrulline (A and C) and internal standard L-citrulline–d6 (B and D) extracted at 159.10 and 165.12 Da corresponding to the fragments of L-citrulline and L-citrulline-d6 respectively. Chromatograms are shown for L-citrulline following incubations at 0 µM ADMA (A) and 45 µM ADMA (C). The corresponding internal standard chromatograms are shown in panels B and D.


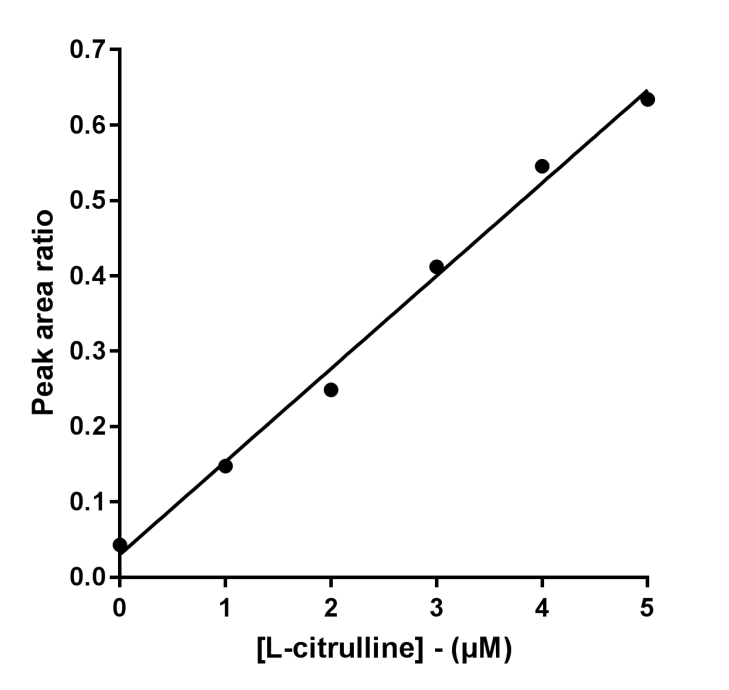


**Figure S2.** L-citrulline calibration curves were constructed by plotting the peak area ratio L-citrulline to internal standard versus the L-citrulline concentration. Each data point represents the mean of two injections.

**DDAH1 activity assay**

L-Citrulline formation was determined at 37 °C in a total incubation volume of 0.1 mL using 12 x 75 mm borosilicate glass tubes. Incubation mixtures contained HEK293T cell lysate expressing recombinant human DDAH1 (0.4 mg/mL), phosphate buffer (0.1 M, pH 7.4) and ADMA (0 to 500 μM).

Following a 5-min period pre-incubation reactions were initiated by the addition of substrate (ADMA). After 30-min incubation the reaction was terminated by the addition of 300 μL 0.1% formic acid in 2-propanol and 10 µL of the assay internal standard (30 µM L-citrulline-d6). The samples were vortex mixed (20 sec) and cooled on ice for 10 min prior to centrifugation (10 min, 18,000 x g) to precipitate the proteins. 300 μL of the supernatant layer was transferred to clean 12 x 75 mm borosilicate glass tubes and the solvent removed by evaporation in a MiVac concentrator (T=50°C, P= 30 mbar, -OH programme, 25 minutes). The residue was redissolved in 125 μL of a 1:4 water / 0.1% formic acid in 2-propanol mixture and a 3 μL aliquot was injected onto the UPLC column for analysis. Samples were maintained at 15 °C in the auto-sampler prior to analysis.

Kinetic constants (*K_m_*, V_max_) for L-citrulline formation were derived from fitting the Michaelis-Menten equation to experimental data using the nonlinear curve fitting software EnzFitter (version 2.0.18.0: Biosoft, Cambridge, UK). Goodness of fit was assessed from the F statistic, 95% confidence intervals, r^2^ value, and standard error of the parameter fit. Kinetic data are the mean of three separate experiments and derived *K_m_* and V_max_ values are reported in Table 1 and Figure 1.

**PPIs and DDAH1 inhibition**

PPIs dissolved in DMSO were added to the DDAH1 Activity Assay such that the final concentration of DMSO in the reaction mixture was 1%. Incubation mixtures comprised of HEK293T cell lysate expressing recombinant human DDAH1 (0.4 mg/mL), phosphate buffer (0.1 M, pH 7.4), PPIs (0, 0.1, 1, 10, 60, 100 μM) and ADMA (45 μM). Following a 5-min pre-incubation of PPI with DDAH1 (37 °C) reactions were initiated by the addition of substrate (ADMA). Protein precipitation and preparation for L-citrulline analysis was performed as described above.

**Effect of pre-incubation time on PPI inhibition of DDAH1**

Time-dependent inhibition of DDAH1 activity by PPIs was characterized by quantification of DDAH1 activity following incubation of each PPI (0 or 60 μM) for 0 (co-incubation), 30, 60, 120, 180, 240 min. The master mix containing DDAH1 (0.4 mg/mL), phosphate buffer (0.1 M, pH 7.4), 1% DMSO and PPI (0 or 60 μM) was pre-incubated in glass tubes at 37 °C. At each time (30, 60, 120, 180, 240 min), 90 µL of the master mix was transferred into a glass tube containing 10 µL of a 10x ADMA stock such that the final concentration of ADMA was 45 μM. The reaction mixture was incubated for a further 30 min at 37 °C. Protein precipitation and preparation for L-citrulline analysis was performed as detailed above.

***
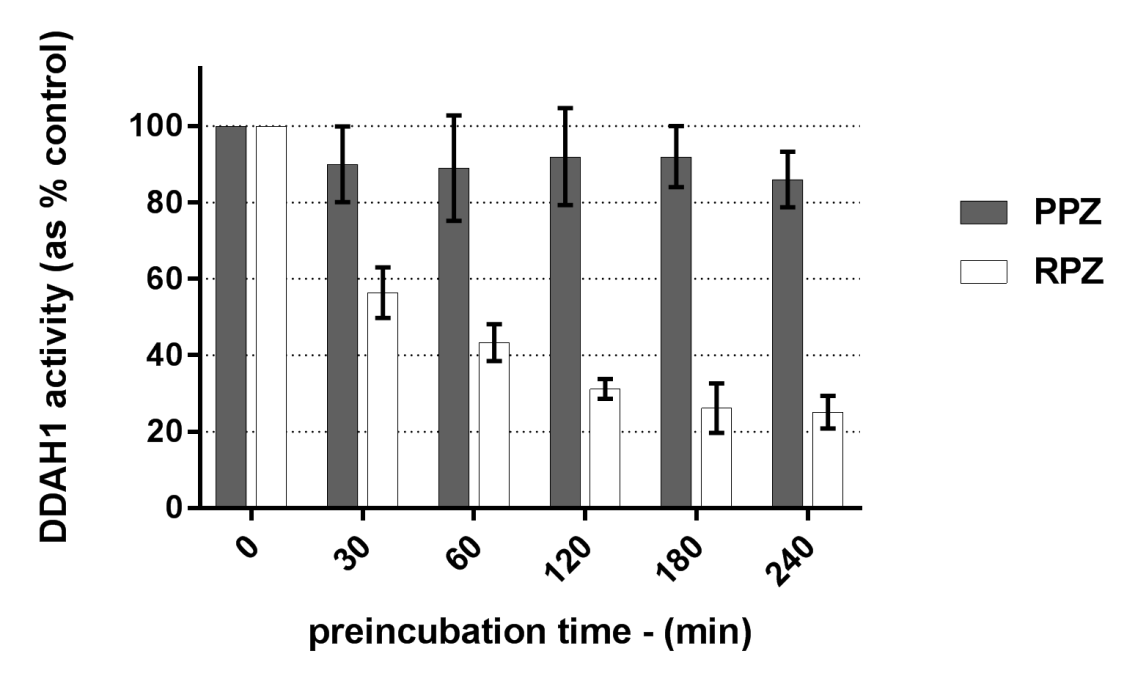
***

**Figure S3.** Time dependent inhibition of DDAH1 by pantoprazole (PPZ) and rabeprazole (RPZ). Each value represents the mean of three singlicate experiments (mean±SD). All experiments were performed with a PPI concentration of 60 µM. Residual DDAH11 activity is expressed as percentage of control (no inhibitor).

**Reversibility**

Reversibility of PPI binding to DDAH1 was assessed by dilution experiments in a conventional two step experimental protocol. DDAH1 (4 mg/mL), phosphate buffer (0.1 M, pH 7.4), 1% DMSO and PPIs (0 or 60 μM) in a total volume of 0.1 mL were incubated at 37 °C. Following a 4-hr incubation a 10 µL aliquot was taken from the incubation mixture and diluted with phosphate buffer (0.1 M, pH 7.4) containing ADMA (final concentration 500 μM) to a final volume of 0.1 mL. L-citrulline formation was assayed to determine DDAH1 enzyme activity as described above.

**Stability of PPIs during incubation**

The stability of PPIs during incubation was determined by incubating each PPI (0 or 60 μM) with DDAH1 (0.4 mg/mL), phosphate buffer (0.1 M, pH 7.4) and 1% DMSO for 4 h at 37 °C. Following addition of ADMA (500 μM final concentration) the reaction mixture was incubated for further 30 min. Proteins were precipitated by addition of 3 volumes of ice-cold methanol to the mixture and cooling on ice for 10 min followed by centrifugation (18,000 x g, 5 min). An aliquot of the supernatant layer was diluted 10-fold with mobile phase and transferred to UPLC vial insert for analysis. Three µL were injected onto the UPLC column for analysis.

For RPZ the time dependent degradation was further investigated at 30, 60, 120 and 180 min. In these experiments samples were diluted 10-fold with 1:1 acetonitrile/water. An incubation containing PPZ instead of RPZ was used as a control.


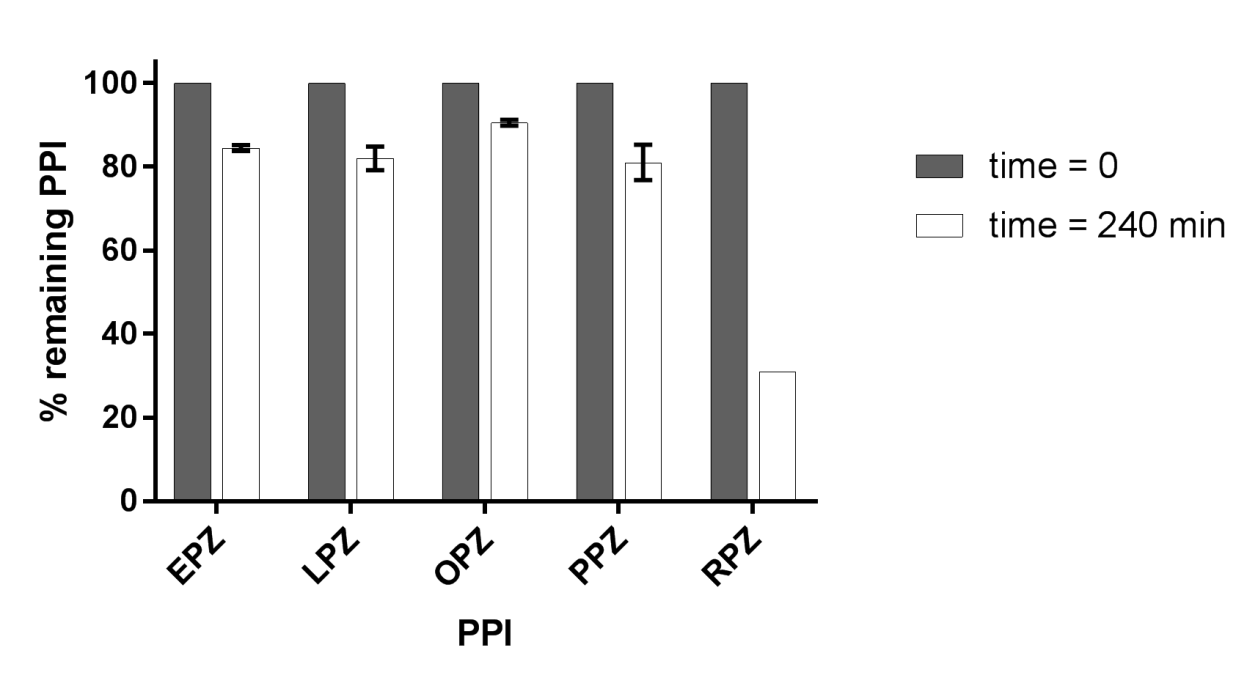


**Figure S4.** PPI stability during incubation. PPI amount is expressed as percentage of control (60 µM PPI at time zero) after 270 min incubation as described above. Each data point represents the mean of duplicate experiments and error bars indicate the % RE for the corresponding measurement.

**Analysis and detection of PPIs and their degradation products**

PPIs were separated from the other components in the mixture on an ACQUITY UPLC® BEH C18 1.7 μm (2.1 x 100 mm) column by isocratic elution at a flow rate of 0.3 mL min^−1^ in a mobile phase comprising 20% of mobile phase A (0.1% formic acid in acetonitrile) and 80% mobile phase B (0.1% formic acid and 10 % acetonitrile in water). The column temperature was maintained at 35 °C whilst the sample compartment was maintained at 20 °C. TUV detection at 280 nm and MS detection in ESI+ V mode were used to detect PPIs and degradation products. Parent PPI’s were detected as [M+H]^+^ at the following mass to charge ratio: EPZ and OPZ 346.09 Da, LPZ 370.05 Da, PPZ 384.04 Da and RPZ 360.11 Da.

Total ion chromatograms were extracted at previously reported m/z and the fragmentation spectra compared to previously reported mass spectra to assign degradation products of RPZ.^17^

**Table S2. Linear regression analysis depicting the association between clinical and demographic characteristics with plasma ADMA concentrations in the Hunter Community Study**

| **Variable** | **B (95% CI)** | **t** | **P-value** |
| --- | --- | --- | --- |
| (Constant) | -0.331 (-0.419 to -0.243) | -7.370 | <0.001 |
| Age (years) | 0.002 (0.001 to 0.003) | 4.495 | <0.001 |
| Gender (female=0, male=1) | -0.012 (-0.025 to 0.001) | -1.854 | 0.064 |
| Regular alcohol consumption (no=0, yes=1) | -0.007 (-0.019 to 0.006) | -1.049 | 0.295 |
| Myocardial infarction (no=0, yes=1) | 0.005 (-0.020 to 0.030) | 0.379 | 0.705 |
| Stroke (no=0, yes=1) | 0.005 (-0.029 to 0.039) | 0.271 | 0.787 |
| Diabetes (no=0, yes=1) | 0.001 (-0.020 to 0.023) | 0.123 | 0.902 |
| Statins (no=0, yes=1) | -0.009 (-0.023 to 0.005) | -1.302 | 0.194 |
| Beta blockers (no=0, yes=1) | 0.018 (0.003 to 0.033) | 2.303 | 0.022 |
| Diuretics (no=0, yes=1) | 0.023 (0.006 to 0.040) | 2.622 | 0.009 |
| Renin-angiotensin system inhibitors (no=0, yes=1) | 0.000 (-0.012 to 0.012) | 0.033 | 0.974 |
| Antiplatelet drugs (no=0, yes=1) | -0.006 (-0.034 to 0.021) | -0.464 | 0.643 |
| Fasting glucose (mmol/L) | -0.005 (-0.012 to 0.001) | -1.663 | 0.097 |
| LDL-cholesterol (mmol/L) | -0.002 (-0.010 to 0.006) | -0.507 | 0.613 |
| HDL-cholesterol (mmol/L) | -0.007 (-0.026 to 0.012) | -0.687 | 0.493 |
| Triglycerides (mmol/L) | 0.006 (-0.003 to 0.015) | 1.413 | 0.158 |
| C-reactive protein (mg/L) | 0.000 (-0.001 to 0.001) | 0.284 | 0.776 |
| Estimated glomerular filtration rate (mL/min/1.73 m^2^) | 0.000 (-0.001 to 0.000) | -1.413 | 0.158 |
| Proton pump inhibitors (no=0, yes=1) | 0.012 (-0.001 to 0.025) | 1.770 | 0.077 |
